# Household survey on prevalence and risk factors for obesity in owned cats from Central Brazil

**DOI:** 10.1371/journal.pone.0337397

**Published:** 2026-01-07

**Authors:** Danilo Conrado Silva, Mariana Yukari Hayasaki Porsani, Aparecido Divino da Cruz, Erika Figueiredo Pereira, Klayto José Gonçalves dos Santos, Lysa Bernardes Minasi, Vitória Alvarenga Nunes, Alex Silva da Cruz, Fabio Alves Teixeira

**Affiliations:** 1 Veterinary Medicine, West Campus, State University of Goiás, São Luís de Montes Belos, Goiás, Brazil; 2 National Association of São Paulo for Small Animal Practice (ANCLIVEPA-SP), São Paulo, Brazil; 3 Replicon Research Group, Genetics Graduate Program, School of Medical and Life Sciences, Pontifical Catholic University of Goiás, Goiânia, Goiás, Brazil; 4 School of Veterinary Medicine and Animal Science, University of São Paulo, São Paulo, Brazil; 5 Center for Animal Reproduction Biotechnology (Biotec), Graduate Program in Animal Production and Forage Science, West Campus, State University of Goiás, São Luís de Montes Belos, Goiás, Brazil; University of Life Sciences in Lublin, POLAND

## Abstract

Few studies have conducted the prevalence of obesity in cat populations and the associated risk factors by assessing cats in their homes, regardless of whether they had visited a veterinary hospital. Moreover, such studies are scarce in Latin America, and, to date, few census-based, in-home epidemiological assessment of feline obesity has been conducted in Brazil. This study aimed to estimate the prevalence of obesity in owned cats in the metropolis of Goiânia, Goiás, Brazil, and to assess the presence of risk factors related to the animals, their owners, the home environment, and cat management practices. A cross-sectional study was conducted with 188 cats and their owners, using geographically stratified household sampling. The animals were categorized based on body condition score (BCS) as underweight, ideal weight, overweight, or obese, and prevalence rates were calculated. The BCS was investigated for its association with animal, owner, environmental, and management characteristics using the Kruskal-Wallis test (α = 5%). Subsequently, characteristics with p ≤ 0.05 were analyzed as potential risk factors for overweight or obesity using binary logistic regression. Kappa analysis was used to determine the agreement between the BCS determined by veterinarians and the owners’ perception of their cats’ body condition. The prevalence of overweight and obesity in cats was 28.7%. Factors related to age and activity level, type of household, as well as the owners’ education and occupation, were identified as risk factors for the development of overweight or obesity in the study cat population. Additionally, protective factors against overweight and obesity were identified, including the number of cats in the household, the location of the household, and the owners’ perception of their own diet. The agreement between the actual BCS of the animals and the owners’ perception of their cats’ body condition was considered low.

## Introduction

Obesity is the most common nutritional disorder in small animal veterinary clinical practice [1,2]. Excessive food intake causes the accumulation of excessive body fat, leading to numerous health problems and reduced life expectancy. Obesity in cats is associated with genetic, environmental, behavioral, sociocultural factors, and even factors directly related to their owners, such as socioeconomic status, eating habits, age and body condition [1–3].

Obesity is a multifactorial disease, although it is closely related to diet and feeding management that lead to a positive energy balance [4]. In veterinary medicine, risk factors for obesity inherent to animals are also described, such as breed, age, sex, reproductive status, hormonal influences, and low physical activity [2,5].

In the United Kingdom, United States, New Zealand, Australia, France, and the Netherlands, the prevalence of overweight and obesity in cats has been estimated to range from 11.5% to 63% [6–15], showing a large variation due to diverse population conditions, as well as methodological and sampling variations. These studies identified potential risk factors for the development of obesity in cats, including breed, age, sex, reproductive status, relationship with the owner, owners’ perception of their cats’ body condition, diet type, feeding frequency, and environment.

Few publications on the prevalence of feline obesity in Brazil were identified, but their findings are discrepant. A study conducted in a small town (Alegre, Espírito Santo), which involved household visits, reported a prevalence of overweight cats at 14%, with 6% classified as obese [16]. In contrast, a study carried out in a major metropolitan area, in the city of Rio de Janeiro, found that nearly 60% of cats presenting to a veterinary hospital for surgical procedures were overweight, with 36.8% classified as obese [17]. Finally, in the southern region of the country (Porto Alegre, Rio Grande do Sul), another study evaluating cats seen at a veterinary hospital—specifically during the COVID-19 pandemic—found yet another different value: 38.4% of cats were overweight, with only 8.7% classified as obese [18].

The reported risk factors for feline obesity were largely consistent across both studies [16,17]. Middle-aged, neutered male cats, particularly those living in households with free access to food, were more likely to be obese. Additionally, owner underestimation of the cat’s body condition score (BCS) contributed as a predisposing factor [17]. In both studies, male sex and neutering were associated with increased obesity risk, whereas physical activity was identified as a protective factor [16].

Despite prevalence studies and risk factors related to obesity in cats having been conducted in different locations around the world, data are scarce in Latin America, and there is limited data from Brazil, especially using a census-based approach with home visits. Therefore, the aim of this study was to estimate the prevalence of obesity in owned cats in the city of Goiânia, state of Goiás, Brazil, and to examine risk factors related to the animals, their owners, the home environment, and cat management practices.

## Materials and methods

### Sample group and ethical considerations

A cross-sectional study was conducted in the city of Goiânia, Brazil, with owned cats as the experimental units to determining the prevalence of obesity and associated risk factors. In addition, data were collected from the animals and their owners, as well as from management practices. The study was conducted between March and September of 2023. This study was approved by the Animal Ethics Committee (approval number 014/2022) and the Research Ethics Committee (approval number 5.925.047) of the State University of Goiás. Data collection was performed after the pet owners included in the study signed the informed consent form.

The minimum number of animals required to determine the prevalence of obesity and associated risk factors was defined using the sample size equation (n) based on the expected prevalence (EP), where n = EP x (1 – EP) x (1.96)²/ 0.052. An EP of 14% for obesity in cats was considered, based on the only study that estimated the prevalence of owned cats through home visits sampling in Brazil [6]. A margin of error of 5% and a 95% confidence level were used. Therefore, the minimum sample size was calculated, resulting in 185.01, thus minimum n = 186 cats. Cats under 1 year of age were excluded from the study due to the focus on assessing the prevalence of obesity in adult cats.

Sampling continued until two of the following criteria were met. The first criterion was a minimum of 186 cats. The second criterion was household sampling through geographic stratification, ensuring that the proportion of households in each weighting area of the city of Goiânia was approximately maintained [19]: South (19.7%), Central (18.8%), Southwest (16.4%), East (12.1%), Northwest (11.3%), West (11.2%), and North (10.5%).

Before sampling in each weighting area, neighborhoods and streets to be visited were randomly selected. The first household visited in each street was always the one with the lowest number, continuing to the immediately adjacent house if sampling could not be conducted for any reason. In households with more than one cat, all cats were evaluated. In cases where two or more owners were present, only the one most involved in the animals’ management was assessed.

### Data collection and categorization of cats and owners

Each selected owner completed a questionnaire to collect personal and family data, as well as information about the cats and their management (Box 1). Owner data included eating habits, physical activity, and socioeconomic status. Owners’ eating habits were classified according to the Dietary Guidelines for the Brazilian Population [20], with “unhealthy” eating habits defined as consuming snacks three or more times per week or consuming fruits and vegetables two or fewer times per week. For the purpose of stratifying owners by household income, amounts in the Brazilian currency, the Brazilian real, were converted into US dollars.

**Box 1. Questionnaire answered by owners included in the study**

**Table:** 

**1 – Your age** a) 18–24 years b) 25–34 years c) 35–44 years d) 45–59 years e) 60–75 years f) Older than 76 years	**2 – Gender** a) Male b) Female **3 – The house where you live is?** a) Owned b) Rented c) Tenant without a lease	**4 – Education** a) Elementary School b) Middle School c) High School d) Collage/University e) Specialization f) Did not attend school
**5 – Total monthly household income** a) No income b) Up to $ 225 c) From $ 225 to $ 670 d) From $ 670 to $ 1.790 e) More than $ 1.790	**6 – Employment Status** a) Government (Public Sector) b) Company (Private or State) c) Non-governmental organization d) Self-employed e) Rural Property f) Unemployed g) Retired h) Homemaker i) Student	**7 – How many weekly hours do you work?** a) No set work hours, up to 10 hours per week b) From 11 to 30 hours per week c) From 30 to 40 hours per week d) More than 40 hours per week
**8 – Number of people that live with you and their age (including employees)** a) Between 0 and 12 years b) Between 13 and 18 years c) Between 19 and 24 years d) Between 25 and 34 years e) Between 35 and 44 years f) Between 45 and 59 years g) Between 60 and 75 years h) Older than 76 years	**9 – Your physical activity frequency** a) Daily b) Once a week c) Three times a week d) More than three times a week e) Does not exercise	**10 – Do you believe you eat** a) In excess b) Normally c) Low amounts **11 – How many times do you consume fried food?** a) Daily b) 1–2 times a week c) 3 or more times a week d) Occasionally e) Never
**12 – How many times do you consume fruits?** a) Daily b) 1–2 times a week c) 3 or more times a week d) Occasionally e) Never	**13 – How many times do you consume vegetables?** a) Daily b) 1–2 times a week c) 3 or more times a week d) Occasionally e) Never	**14 – How many times do you consume snacks?** a) Daily b) 1–2 times a week c) 3 or more times a week d) Occasionally e) Never

Data on the animals included basic information such as age, sex, breed, and reproductive status, as well as sanitary and nutritional management practices. Additionally, the owner’s perception of their cats’ current body condition was also collected. Owners chose their cats’ body condition by selecting one of the following categories in the questionnaire: underweight, ideal weight, overweight, or obese.

The cats were evaluated by veterinarians using the nine-point BCS [21] and classified as underweight (BCS 1–4), ideal weight (BCS 5), overweight (BCS 6–7), and obese (BCS 8–9). Age classification followed the Feline Life Stage Guidelines [22]: 1–6 years – young adult; 7–10 years – mature adult; over 10 years – senior.

Owner’s body mass index (BMI) was calculated using the methodology recommended by the World Health Organization [23] through the equation: BMI = weight (kg)/ height² (m²). Height was measured with a tape measure, and weight was measured on a portable digital scale. BMI was classified as underweight (<18.5), normal weight (18.6–24.9), overweight (25.0–29.9), and obesity (≥30.0). Anthropometric measurements were also taken using a tape measure, including abdominal, waist and hip circumferences. A waist/height ratio of >0.52 was considered a potential health risk, while waist/hip ratios of <0.91 for men and <0.76 for women were considered low risk for cardiovascular disease [24].

### Statistical analysis

The results were presented and analyzed through frequencies distributed among the determined cats’ BCS (underweight, ideal weight, overweight, and obesity), as well as the characteristics of the animals (intrinsic and sanitary characteristics), the owners (intrinsic, anthropometric, lifestyle habits, socioeconomic characteristics, and perception of the animals’ body condition), and the cats’ dietary management.

To assess potential differences in the distribution of BCS frequencies across each characteristic analyzed, the Kruskal-Wallis test was performed with a significance level of 5%. Subsequently, characteristics with p ≤ 0.05 were analyzed as potential risk factors for being overweight or obesity using binary logistic regression (BLR), from which odds ratios (OR) and 95% confidence intervals were calculated.

Kappa analysis was used to determine agreement between BCS determined by veterinarians and the owners’ perception of their cats’ body condition. The agreement is considered low if between 0.00 and 0.20; reasonable if between 0.21 and 0.40; moderate if between 0.41 and 0.60; high if between 0.61 and 0.80; and almost perfect if between 0.81 and 1.00 [1].

All statistical analyses were conducted using statistical software (SPSS, version 20; IBM Corporation).

## Results and discussion

This study included 188 cats from 80 households, with an average of 2.35 cats per household. A total of 1043 households were visited, but 963 were excluded due to various reasons: no residents present at the time of the visit (547), households without cats (318), aggressive or missing cats (71), and owners who decline to participate (27).

The 80 households included in the study were sampled across the seven weighting areas of Goiânia, distributed as follows: 16 in the South (20%), 15 in the Central (18.75%), 13 in the Southwest (16.25%), 10 in the East (12.5%), 9 in the Northwest (11.25%), 9 in the West (11.25%), and 8 in the North (10%).

The BCS assessment classified 57 cats (30.3%) as underweight, 77 (41%) as ideal weight, 38 (20.2%) as overweight, and 16 (8.5%) as obese. Thus, the total prevalence of overweight and obese cats was 28.7%, with 20.2% classified as overweight and 8.5% as obese. Studies from various countries have estimated the prevalence of obesity in cats across different populations, with overweight and obesity rates ranging from 11.5% to 63% [6–15]. According to Tarkosova et al. [25], this wide variation in prevalence can be attributed to the specific characteristics of each studied population such as geographic region, differences in feeding practices, and the proportion of neutered or indoor cats.

Our values are intermediate compared to the prevalence rates of overweight and obesity reported in other Brazilian studies, which ranged from 14% to 38.4% [16–18]. They are more similar to the only study that also conducted home visits, which found 6% of cats to be obese [16]. Despite the similarity, this study was carried out in a city with a human population approximately 50 times smaller than that of Goiânia [19].

The distribution of BCS categories was compared with the intrinsic characteristics of the cats in the study (Table 1). Differences (p < 0.05) in distribution of BCS frequencies across each characteristic were observed based on age group, reproductive status, age at neutering, and the level of physical activity reported by the owner.

**Table 1:** Distribution of body condition score (BCS) frequencies accordingly with each intrinsic characteristic of owned cats in Goiânia, Brazil.

Intrinsic Characteristic	Underweight (BCS 1–4)		Ideal (BCS 5)		Overweight (BCS 6–7)		Obese (BCS 8–9)		Total		p-value*
	N	(%)	N	(%)	N	(%)	N	(%)	N	(%)	
**Sex**											
Male	25	(32.1)	34	(43.6)	10	(12.8)	9	(11.5)	78	(41.5)	0.147
Female	32	(29.1)	43	(39.1)	28	(25.4)	7	(6.4)	110	(58.5)	
**Age range**											
1 to 6 years	37	(27.2)	64	(47.1)	28	(20.6)	7	(5.1)	136	(74.3)	0.009
7 to 10 years	12	(34.3)	8	(22.9)	8	(22.9)	7	(20.0)	35	(19.1)	
More than 10 years	4	(33.3)	4	(33.3)	2	(16.7)	2	(16.7)	12	(6.6)	
**Reproductive status**											
Neutered	36	(24.3)	63	(42.6)	33	(22.3)	16	(10.8)	148	(78.7)	0.002
Intact	21	(52.5)	14	(35.0)	5	(12.5)	0	(0.0)	40	(21.3)	
**Sex and reproductive status**											
Neutered male	14	(25.0)	25	(44.6)	8	(14.3)	9	(16.1)	56	(29.8)	0.084
Intact male	11	(50.0)	9	(40.9)	2	(9.1)	0	(0.0)	22	(11.7)	
Neutered female	22	(23.9)	38	(41.3)	25	(27.2)	7	(7.6)	92	(48.9)	
Intact female	10	(55.5)	5	(27.8)	3	(16.7)	0	(0.0)	18	(9.6)	
**Age at neutering**											
Up to 1 year	5	(27.8)	3	(16.7)	6	(33.3)	4	(22.2)	18	(27.3)	0.005
More than 1 year	3	(37.5)	1	(12.5)	3	(37.5)	1	(12.5)	8	(12.1)	
Intact cats	24	(52.5)	16	(35.0)	6	(12.5)	1	(0.0)	47	(60.6)	
**Breed**											
Mixed breed	57	(31.5)	74	(40.9)	36	(19.9)	14	(7.7)	181	(96.3)	0.118
Purebred	0	(0.0)	3	(43.0)	2	(28.5)	2	(28.5)	7	(3.7)	
**Physical activity**											
Active	38	(31.9)	55	(46.2)	20	(16.8)	6	(5.1)	119	(63.3)	0.031
Inactive	19	(27.5)	22	(31.9)	18	(26.1)	10	(14.5)	69	(36.7)	

BCS: Body Condition Score; *p value obtained by the Kruskal-Wallis test.

In contrast to previous studies that identified association between obesity and male cats [8,10,11,15–17], it was not found in the present study, despite the higher frequency of obese males (Table 1). Neutering has been described in the literature as an important risk factor for overweight and obesity in cats [7,8,10,11,13,17,26]. In the present study, despite the reproductive status statistically influencing the cats’ BCS (Table 1) and reproductive status seems to influence the cats’ BCS due to the higher frequency of intact cats with a BCS < 4, neutering was not a risk factor for overweight or obese cats (p = 0.161) (Table 2). A recent study [26] discusses the relationship between the age at neutering and its effect on feline body condition. In our data, the distribution of body condition score (BCS) also differed according to the age at neutering (Table 1).

**Table 2:** Binary logistic regression analysis of variables statistically significant in the Kruskal-Wallis test associated with overweight and obese body condition of owned cats in Goiânia, Brazil.

Characteristic	Ideal (BCS 5)		Overweight and obese (BCS ≥ 6)		Total		OR	Cl-95%	p-value*
	N	%	N	%	N	%			
**Age range**									
1 to 6 years	64	(64.6)	35	(35.4)	99	(100)	3.5	1.4-9.1	0.008
7 to 10 years	8	(34.8)	15	(65.2)	23	(100)			
**Reproductive status**									
Neutered	63	(56.3)	49	(43.7)	112	(100)	0.459	0.15-1.4	0.161
Intact	14	(73.7)	5	(26.3)	19	(100)			
**Physical activity**									
Active	55	(67.9)	26	(32.1)	81	(100)	2.7	1.3-5.6	0.008
Inactive	22	(44.0)	28	(56.0)	50	(100)			
**Vaccinated**									
Yes	66	(57.9)	48	(42.1)	114	(100)	0.75	0.3-2.2	0.595
No	11	(64.7)	6	(35.3)	17	(100)			
**Vaccination frequency**									
Annual	47	(59.5)	32	(40.5)	79	(100)	1.2	0.5-2.8	0.604
Overdue	19	(54.3)	16	(45.7)	35	(100)			
**Has been assessed by a veterinarian**									
Yes	21	(70.0)	9	(30.0)	30	(100)	0.5	0.2-1.3	0.159
No	56	(55.4)	45	(44.6)	101	(100)			
**Frequency of visits to a veterinarian**									
Annual	23	(54.8)	19	(45.2)	42	(100)	1	0.5-2.2	0.981
Only when sick	33	(55.0)	27	(45.0)	60	(100)			
**Disease status reported by the owner**									
Yes	23	(69.7)	10	(30.3)	33	(100)	2.8	0.9-8.5	0.076
No	10	(45.5)	12	(54.5)	22	(100)			
**Type of household**									
House	76	(62.3)	46	(37.7)	122	(100)	13.2	1.6-109.1	0.017
Apartament	1	(11.1)	8	(88.9)	9	(100)			
**Presence of other cat(s) in the household**									
Yes	65	(61.9)	40	(38.1)	105	(100)	1.9	0.8-4.5	0.148
No	12	(46.2)	14	(53.8)	26	(100)			
**Presence of dog(s) in the household**									
Yes	29	(61.7)	18	(38.3)	47	(100)	1.2	0.6-2.5	0.611
No	48	(57.1)	36	(42.9)	84	(100)			
**Number of cats in the household**									
1–4	31	(43.7)	40	(56.3)	71	(100)	0.2	0.1-0.5	<0.0001
5 or more	46	(76.7)	14	(23.3)	60	(100)			
**Education Level**									
No formal education or up to middle school	21	(84.0)	4	(16.0)	25	(100)	4.7	1.5-14.6	0.008
High school to postgraduate education	56	(52.8)	50	(47.2)	106	(100)			
**Owner’s occupation**									
Home	50	(68.5)	23	(31.5)	73	(100)	2.5	1.2-5.1	0.012
Outside of home	27	(46.5)	31	(53.5)	58	(100)			
**Household income**									
Up to $ 670	52	(65.0)	28	(35.0)	80	(100)	1.9	0.9-3,9	0.072
More than $ 670	25	(49.0)	26	(51.0)	51	(100)			
**City region**									
Other regions	56	(69.1)	25	(30.9)	81	(100)	0.3	0.1-0.7	0.003
South/East/North	21	(42.0)	29	(58.0)	50	(100)			
**Perception of the owner’s eating habits**									
Normal/In excess	71	(62.8)	42	(37.2)	113	(100)	0.3	0.1-0.8	0.023
Low amounts	6	(33.3)	12	(66.7)	18	(100)			

BCS: Body Condition Score; OR: odds ratio; CI-95%: 95% confidence interval. *P-value obtained by the binary logistic regression.

Middle-aged cats, between 7 and 10 years old, had the highest frequency of overweight and obesity (Table 1) and OR = 3.5 (Table 2), supporting the literature [9–11,14,17,27], that showed the prevalence of obesity in cats tends to increase up to 10 years of age and then decreases.

The activity levels of the cats reported by their owners influenced the cats’ BCS (Table 1). Greater inactivity was shown to be a risk factor for overweight and obesity (OR = 2.7; Table 2). Increased opportunities for physical activity have already been described as a protective factor against obesity in cats [16,28]. In a cross-sectional study conducted in New Zealand [12], cats were categorized as inactive, normal, or hyperactive, with the prevalence of overweight/obesity found to be 81%, 63%, and 46%, respectively (p = 0.037).

The breed was not statistically associated with BCS (Table 2), which may be explained by the small sample size of purebreds cats. According to Courcier et al. [11], breed was not considered a risk factor for overweight or obesity in cats in Great Britain. However, other studies have shown a higher susceptibility in certain breeds as Norwegian Forest Cat, British Shorthair and Persian [9,13] or in cats with no defined breed [8,15,27].

In Brazil, the frequency of overweight and obesity was also assessed in two samples populations of cats visiting veterinary hospitals. A population of 106 cats undergoing surgical procedures at a feline private veterinary clinic in Rio de Janeiro [17] and a population of 389 cats visiting a teaching hospital in Porto Alegre [18]. Among the evaluated cats, 60.4% and 38.4% were above ideal weight, with 23.6% and 29.7% classified as overweight, and 36.8% and 8.7% as obese, respectively. The frequency of overweight and obesity in the first study was considerably higher than the estimated prevalence in Goiânia, while the second was similar for obesity but with higher occurrence of overweight cats. In this context, the discrepancies among the four studies conducted on feline obesity prevalence may be attributed to methodological differences, as two were based on hospital-attending populations and two employed a census-based home visit model. Although the latter is theoretically more representative and reliable, it is inherently influenced by the regional characteristics of the sampled population. In the present study, for instance, the number of veterinary visits showed a statistically significant association with obesity prevalence, as identified by the Kruskal-Wallis test.

Regarding the sanitary aspects evaluated, differences in BCS distribution were observed between vaccination status, vaccination frequency, veterinary visits, and their frequency, as well as the diseases status reported by the owner (Table 3). Lack of or delayed vaccination, as well as not visiting the veterinarian, were associated with higher prevalence of underweight cats, which may have been due to a higher frequency of diseases in these groups. In our sample, this theory was supported by the higher prevalence of underweight cats that, according to the owners, were ill, although none of these sanitary characteristics were considered risk factors for obesity (Table 2). Similarly, it can be hypothesized that animals with more frequent veterinary visits tend to receive greater overall care from their owners, which may include the provision of higher-quality diets—typically characterized by greater digestibility and caloric density. These are likely indoor cats with limited space for physical activity, that are neutered and have greater longevity. Therefore, these factors may explain why the frequency of veterinary visits is associated with the distribution of body condition scores and the observed differences in the prevalence of overweight and obesity across this data and other Brazilian studies [16–18].

**Table 3:** Distribution of body condition score (BCS) frequencies according to the sanitary characteristics of owned cats in Goiânia, Brazil.

Sanitary Characteristics	Underweight (BCS 1–4)		Ideal (BCS 5)		Overweight (BCS 6–7)		Obese (BCS 8–9)		Total		p-value*
	N	%	N	%	N	%	N	%	N	%	
**Vaccinated**											
Yes	36	(24.0)	66	(44.0)	33	(22.0)	15	(10.0)	150	(79.8)	0.002
No	21	(55.3)	11	(28.9)	5	(13.2)	1	(2.6)	38	(20.2)	
**Vaccination frequency**											
Annual	20	(20.2)	47	(47.4)	23	(23.3)	9	(9.1)	99	(52.7)	0.003
Overdue	16	(31.4)	19	(37.2)	10	(19.6)	6	(11.8)	51	(27.1)	
Never	21	(55.3)	11	(28.9)	5	(13.2)	1	(2.6)	38	(20.2)	
**Vaccination in a public rabies campaign**											
No	36	(30.2)	48	(40.3)	24	(20.2)	11	(9.2)	119	(63.3)	0.972
Yes	21	(30.4)	29	(42.0)	14	(20.3)	5	(7.3)	69	(36.7)	
**Vaccinated by veterinarian**											
No	24	(38.7)	22	(35.5	10	(16.1)	6	(9.7)	62	(33.0)	0.294
Yes	33	(26.2)	55	(43.7)	28	(22.2)	10	(7.9)	126	(67.0)	
**Has been assessed by a veterinarian**											
No	26	(46.4)	21	(37.5)	6	(10.7)	3	(5.4)	56	(29.8)	0.009
Yes	31	(23.5)	56	(42.4)	32	(24.2)	13	(9.9)	132	(70.2)	
**Frequency of visits to a veterinarian**											
Annual	14	(25.0)	23	(41.1)	14	(25.0)	5	(8.9)	56	(29.8)	0.035
Only when sick	17	(22.1)	33	(42.9)	18	(23.3)	9	(11.7)	77	(41.0)	
Never	26	(47.3)	21	(38.2)	6	(10.9)	2	(3.6)	55	(29.2)	
**Disease reported by the owner**											
No disease	32	(24.6)	54	(41.6)	32	(24.6)	12	(9.2)	130	(69.1)	0.129
Infectious or parasitic disease	20	(54.0)	14	(37.9)	2	(5.4)	1	(2.7)	37	(19.7)	
Orthopedic disease	3	(27.3)	5	(45.5)	2	(18.1)	1	(9.1)	11	(5.9)	
Urinary disease	1	(25.0)	1	(25.0)	2	(50.0)	0	(0.0)	4	(2.1)	
Neoplasia	0	(0.0)	2	(100)	0	(0.0)	0	(0.0)	2	(1.1)	
Intoxication	1	(50.0)	0	(0.0)	0	(0.0)	1	(50.0)	2	(1.1)	
Gastritis	0	(0.0)	1	(100)	0	(0.0)	0	(0.0)	1	(0.5)	
Diabetes mellitus	0	(0.0)	0	(0.0)	0	(0.0)	1	(100)	1	(0.5)	
**Disease status reported by the owner**											
Yes	25	(43.1)	23	(39.7)	6	(10.3)	4	(6.9)	58	(30.8)	0.043
No	8	(26.7)	10	(33.3)	10	(33.3)	2	(6.7)	30	(69.2)	
**FIV and/or FeLV reported by owners**											
Yes	8	(47.0)	7	(41.2)	1	(5.9)	1	(5.9)	17	(19.3)	0.215
No	25	(35.2)	26	(36.6)	15	(21.1)	5	(7.1)	71	(80.7)	

BCS: Body Condition Score; *p value obtained by the Kruskal-Wallis test.

Chiang et al. [17] reported a higher prevalence of BCS between 1 and 5 in cats with immune/infectious diseases. In the same study, dental, orthopedic, and urinary diseases were positively associated with overweight and obesity. Lund et al. [10] found a higher prevalence of overweight or obese cats with oral cavity and urinary tract diseases. In our study, however, the specific diseases reported by owners did not influence the BCS distribution of the animals.

None of the feeding management characteristics were statistically associated with the cats’ BCS (Table 4). Supporting these findings, another study conducted in Brazil with owned cats in a smaller city also found no correlation between feeding frequency or type of food provided and obesity development [16]. Studies conducted in New Zealand [6] and France [10] also did not identify feeding management factors as risk factors for obesity. On the other hand, food quantity [28], free access to food [7,17], commercial dry diets [27,28], and the lack of food portion control [27] have been described as risk factors for the development of overweight or obesity in cats.

**Table 4:** Distribution of body condition score (BCS) according to the feeding management of owned cats in Goiânia, Brazil.

Feeding management	Underweight (BCS 1–4)		Ideal (BCS 5)		Overweight (BCS 6–7)		Obese (BCS 8–9)		Total		p-value*
	N	%	N	%	N	%	N	%	N	%	
**Type of diet**											
Commercial diets (wet and dry)	33	(25.2)	55	(42.0)	29	(22.1)	14	(10.7)	131	(69.7)	0.064
Commercial diets (wet and dry) and homemade diet	23	(41.1)	22	(39.3)	9	(16.1)	2	(3.5)	56	(29.8)	
Only homemade diet	1	(100)	0	(0.0)	0	(0.0)	0	(0.0)	1	(0.5)	
**Feeding frequency**											
Once a day	0	(0.0)	0	(0.0)	1	(100)	0	(0.0)	1	(0.5)	0.640
Twice a day	17	(37.8)	20	(44.4)	4	(8.9)	4	(8.9)	45	(24.0)	
Three times a day	6	(17.1)	17	(48.6)	9	(25.7)	3	(8.6)	35	(18.6)	
*Ad libitum*	34	(31.8)	40	(37.4)	24	(22.4)	9	(8.4)	107	(56.9)	
**Method of quantification of daily food intake**											
Measuring cup	4	(23.5)	7	(41.2)	5	(29.4)	1	(5.9)	17	(9.0)	0.750
Does not quantify	53	(31.0)	70	(40.9)	33	(19.3)	15	(8.8)	171	(91.0)	
**Criteria to determine daily food intake**											
Veterinarian	0	(0.0)	0	(0.0)	1	(100)	0	(0.0)	1	(0.5)	0.731
Owner choice	4	(25.0)	7	(43.7)	4	(25.0)	1	(6.3)	16	(8.5)	
No information	53	(31.0)	70	(40.9)	33	(19.3)	15	(8.8)	171	(91.0)	
**Gives treats**											
Yes	37	(31.9)	47	(40.5)	24	(20.7)	8	(6.9)	116	(61.7)	0.749
No	20	(27.8)	30	(41.7)	14	(19.4)	8	(11.1)	72	(38.3)	
**Type of treats**											
Cat treats	13	(28.3)	18	(39.1)	11	(23.9)	4	(8.7)	46	(24.4)	0.725
Human food	6	(40.0)	6	(40.0)	2	(13.3)	1	(6.7)	15	(8.0)	
Cat treats and human food	18	(32.7)	23	(41.8)	11	(20.0)	3	(5.5)	55	(29.3)	
Does not give treats	20	(27.8)	30	(41.7)	14	(19.4)	8	(11.1)	72	(38.3)	

BCS: Body Condition Score; *p value obtained by the Kruskal-Wallis test.

The type of household had a statistically significant influence on cats’ BCS (p = 0.003). The prevalence of overweight or obese cats was higher in the apartment group (Table 5). Living in an apartment was identified as a risk factor for overweight and obesity (OR = 13.2) (Table 2). Partial or total restriction to outdoor environments has also been reported as a risk factor for obesity in cats in other studies [14,27,28].

**Table 5:** Distribution of body condition score (BCS) frequencies according to household characteristics of owned cats in Goiânia, Brazil.

Household characteristics	Underweight (BCS 1–4)		Ideal (BCS 5)		Overweight (BCS 6–7)		Obese (BCS 8–9)		Total		p-value*
	N	%	N	%	N	%	N	%	N	%	
**Type of household**											
House	56	(31.5)	76	(42.7)	33	(18.5)	13	(7.3)	178	(94.7)	0.003
Apartament	1	(10.0)	1	(10.0)	5	(50.0)	3	(30.0)	10	(5.3)	
**Other pets in the household**											
Cats	29	(29.6)	41	(41.8)	20	(20.4)	8	(8.2)	98	(52.1)	0.016
Dogs	0	(0.0)	5	(45.4)	4	(36.4)	2	(18.2)	11	(5.8)	
Dogs and cats	25	(41.0)	24	(39.3)	8	(13.1)	4	(6.6)	61	(32.5)	
No other animals	3	(16.7)	7	(38.9)	6	(33.3)	2	(11.1)	18	(9.6)	
**Number of cats in the household**											
1	3	(10.3)	12	(41.4)	10	(34.5)	4	(13.8)	29	(15.4)	<0.001
2	5	(20.8)	6	(25.0)	7	(29.2)	6	(25.0)	24	(12.8)	
3 or 4	10	(27.8)	13	(36.1)	9	(25.0)	4	(11.1)	36	(19.1)	
5–10	18	(34.6)	25	(48.1)	7	(13.4)	2	(3.9)	52	(27.7)	
15 or more	21	(44,7)	21	(44.7)	5	(10,6)	0	(0,0)	47	(25,0)	
**Number of people in the household**											
1	12	(33.3)	14	(38.9)	6	(16.7)	4	(11.1)	36	(19.2)	0.107
2	15	(36.6)	14	(34.1)	5	(12.2)	7	(17.1)	41	(21.8)	
3	8	(22.9)	13	(37.1)	12	(34.3)	2	(5.7)	35	(18.6)	
4	11	(30.6)	18	(50.0)	5	(13.9)	2	(5.5)	36	(19.1)	
5	5	(27.8)	9	(50.0)	3	(16.7)	1	(5.5)	18	(9.6)	
6	6	(31.6)	8	(42.1)	5	(26.3)	0	(0.0)	19	(10.1)	
10	0	(0.0)	1	(33.3)	2	(66.7)	0	(0.0)	3	(1.6)	
**Children in the household**											
Yes	16	(33.3)	20	(41.7)	10	(20.8)	2	(4.2)	48	(25.5)	0.652
No	41	(29.3)	57	(40.7)	28	(20.0)	14	(10.0)	140	(74.5)	
**Elderly people in the household**											
Yes	40	(33.3)	51	(42.5)	21	(17.5)	8	(6.7)	120	(63.8)	0.296
No	17	(25.0)	26	(38.2)	17	(24.0)	8	(11.8)	68	(36.2)	

BCS: Body Condition Score; *p value obtained by the Kruskal-Wallis test.

The presence of other pets in the household influenced the distribution of the cats’ BCS (Table 5). However, when analyzed through binary logistic regression, it was not considered a risk factor for overweight or obesity, despite the higher prevalence of overweight or obese cats in homes without other cats (Table 2).

When BCS was evaluated based on the number of cats in the household, it was observed that the presence of at least five cats in the household acted as a protective factor against overweight or obesity (p < 0.001; Table 2). In the study conducted by Cave et al. [12], the number of cats or the presence of dogs in the household did not influence the prevalence of overweight or obesity. Similarly, Colliard et al. [10] did not find a correlation between overweight or obesity and living with other animals. The number of people in the household or the presence of children or elderly individuals did not influence the distribution of BCS in the study cats.

Unlike sex, age group, and work hours, the owners’ education level influenced the cats’ BCS (p = 0.002). A higher prevalence of obese cats was observed among owners with higher education levels (Table 6). Owners with education from high school to postgraduate levels were considered a risk factor for the development of overweight or obesity in the study cats (Table 2).

**Table 6:** Distribution of body condition score (BCS) frequencies according to the intrinsic and socioeconomic characteristics of cat owners in Goiânia, Brazil.

Characteristics	Underweight (BCS 1–4)		Ideal (BCS 5)		Overweight (BCS 6–7)		Obese (BCS 8–9)		Total		p-value*
	N	%	N	%	N	%	N	%	N	%	
**Gender**											
Male	8	(25.8)	15	(48.4)	6	(19.3)	2	(6.5)	31	(16.5)	0.815
Female	49	(31.1)	62	(39.5)	32	(20.4)	14	(9.0)	157	(83.5)	
**Age range**											
18 to 34 years	9	(27.3)	14	(42.4)	8	(24.2)	2	(6.1)	33	(17.6)	0.180
35 to 59 years	18	(23.7)	30	(39.5)	20	(26.3)	8	(10.5)	76	(40.4)	
≥ 60	30	(37.9)	33	(41.8)	10	(12.7)	6	(7.6)	79	(42.0)	
**Education level**											
Did not attend school	0	(0.0)	4	(80.0)	1	(20.0)	0	(0.0)	5	(2.7)	0.002
Elementary School	14	(60.9)	7	(30.4)	2	(8.7)	0	(0.0)	23	(12.2)	
Middle School	3	(21.4)	10	(71.4)	1	(7.2)	0	(0.0)	14	(7.5)	
High School	23	(31.9)	28	(38.9)	17	(23.6)	4	(5.6)	72	(38.3)	
Collage/University	13	(22.8)	23	(40.3)	12	(21.1)	9	(15.8)	57	(30.3)	
Postgraduate	4	(23.5)	5	(29.4)	5	(29.4)	3	(17.7)	17	(9.0)	
**Owner’s occupation**											
Home	37	(33.6)	50	(45.4)	18	(16.4)	5	(4.6)	110	(58.5)	0.028
Outside of home	20	(25.6)	27	(34.7)	20	(25.6)	11	(14.1)	78	(41.5)	
**Work hours**											
Up to10h/week	35	(32.7)	49	(45.8)	18	(16.8)	5	(4.7)	107	(56.9)	0.216
11h to 30h/week	5	(20.8)	7	(29.2)	8	(33.3)	4	(16.7)	24	(12.8)	
30h to 40h/week	10	(40.0)	5	(20.0)	6	(24.0)	4	(16.0)	25	(13.3)	
>40 h/week	7	(21.9)	16	(50.0)	6	(18.7)	3	(9.4)	32	(17.0)	
**Household income**											
Up to $ 225	16	(59.3)	9	(33.3)	2	(7.4)	0	(0.0)	27	(14.4)	0.009
From $ 225 to $ 670	22	(24.2)	43	(47.2)	20	(22.0)	6	(6.6)	91	(48.4)	
From $ 670 to $ 1.790	16	(36.4)	13	(29.5)	10	(22.7)	5	(11.4)	44	(23.4)	
More than $ 1.790	3	(11.5)	12	(46.2)	6	(23.1)	5	(19.2)	26	(13.8)	
**City region**											
Central	23	(44.3)	18	(34.6)	10	(19.2)	1	(1.9)	52	(27.7)	0.001
East	2	(10.5)	7	(36.8)	9	(47.4)	1	(5.3)	19	(10.1)	
Northwest	16	(51.6)	12	(38.7)	3	(9.7)	0	(0.0)	31	(16.5)	
North	1	(10.0)	4	(40.0)	2	(20.0)	3	(30.0)	10	(5.3)	
West	2	(10.5)	12	(63.2)	4	(21.0)	1	(5.3)	19	(10.1)	
South-west	12	(37.5)	14	(43.7)	4	(12.5)	2	(6.3)	32	(17.0)	
South	1	(4.0)	10	(40.0)	6	(24.0)	8	(32.0)	25	(13.3)	

BCS: Body Condition Score; *p value obtained by the Kruskal-Wallis test.

Household income influenced the cats’ BCS (Table 6). Although household income was not identified as a risk factor for overweight and obesity when analyzed in binary form (Table 2), an increasing prevalence of obese cats was observed as household income increased (Table 6).

The Southern region of Goiânia has a population with a high average household income, according to the literature [29], and in our study, most of the owners living in apartments were concentrated in this area. These factors may explain the high prevalence of obese cats in the southern region. In contrast, the northwestern region of Goiânia, which has a higher concentration of lower-income residents [29], showed the highest prevalence of underweight cats. Excluding the southern, eastern, and northern regions of the city, living in other areas was considered a protective factor against overweight and obesity in the owned cats in Goiânia (Table 2).

The owner’s occupation influenced the cats’ BCS (p = 0.028). Working outside the home was identified as a risk factor for overweight and obesity (OR = 2.5). Although age group did not influence BCS in this study, Colliard et al. [10] found an increased risk of overweight in cats owned by individuals aged 41–60 years.

Owners’ habits related to physical activity, as well as the consumption of fried foods, fruits, vegetables, or snacks did not influence the cats’ BCS (Table 7). In dogs, however, the unhealthy habit of consuming snacks by owners has been identified as a risk factor for the development of overweight and obesity in their pets [1].

**Table 7:** Distribution of body condition score (BCS) frequencies according to the lifestyle habits of cat owners in Goiânia, Brazil.

Characteristics	Underweight (BCS 1–4)		Ideal (BCS 5)		Overweight (BCS 6–7)		Obese (BCS 8–9)		Total		p-value*
	N	%	N	%	N	%	N	%	N	%	
**Physical activity**											
Yes	21	(25.0)	39	(46.4)	15	(17.9)	9	(10.7)	84	(44.7)	0.285
No	36	(34.6)	38	(36.6)	23	(22.1)	7	(6.7)	104	(55.3)	
**Frequency of exercise**											
Does not exercise	36	(34.3)	39	(37.1)	23	(21.9)	7	(6.7)	105	(55.8)	0.262
Once a week	3	(16.7)	11	(61.1)	2	(11.1)	2	(11.1)	18	(9.6)	
Three times a week	6	(24.0)	7	(28.0)	5	(20.0)	7	(28.0)	25	(13.3)	
More than three times a week	5	(41.7)	6	(50.0)	1	(8.3)	0	(0.0)	12	(6.4)	
Daily	7	(25.0)	14	(50.0)	7	(25.0)	0	(0.0)	28	(14.9)	
**Perception of own eating habits**											
In excess	6	(24.0)	15	(60.0)	2	(8.0)	2	(8.0)	25	(13.3)	0.007
Normal	42	(30.9)	56	(41.2)	25	(18.4)	13	(9.5)	136	(72.3)	
Low amounts	9	(33.3)	6	(22.2)	11	(40.8)	1	(3.7)	27	(14.4)	
**Fried food consumption**											
Healthy	37	(26.4)	59	(42.2)	30	(21.4)	14	(10.0)	140	(74.5)	0.193
Unhealthy	20	(41.7)	18	(37.5)	8	(16.7)	2	(4.1)	48	(25.5)	
**Fruit consumption**											
Healthy	52	(32.1)	66	(40.7)	32	(19.8)	12	(7.4)	162	(86.2)	0.389
Unhealthy	5	(19.2)	11	(42.3)	6	(23.1)	4	(15.4)	26	(13.8)	
**Vegetable consumption**											
Healthy	49	(28.3)	72	(41.6)	36	(20.8)	16	(9.3)	173	(92.0)	0.185
Unhealthy	8	(53.3)	5	(33.3)	2	(13.3)	0	(0.0)	15	(8.0)	
**Snack consumption**											
Healthy	42	(30.4)	57	(41.3)	31	(22.5)	8	(5.8)	138	(73.4)	0.123
Unhealthy	15	(30.0)	20	(40.0)	7	(14.0)	8	(16.0)	50	(26.6)	

BCS – Body Condition Score; *p value obtained by the Kruskal-Wallis test.

The owners’ perceptions of their own eating habits were different among different BCS (Table 7). Cats from owners who perceived themselves as eating normal or excessive amounts had a lower prevalence of overweight or obesity (Table 2), with this perception serving as a protective factor. These owners, who considered their food intake to be normal or excessive, appear to have a more accurate perception of their own diet, which may reflect into providing a more appropriate amounts of food for their cats. In contrast, of the 14 owners who claimed to eat low amounts, 8 (57.1%) were overweight or obese, and their cats’ food was not quantified, suggesting a less accurate perception of their own eating habits, which may also be reflected in their pets’ diet.

The anthropometric measurements taken from the owners resulted in the body mass index (BMI) and calculations for waist/hip, waist/height, and abdominal circumference ratios. None of these characteristics were related to the cats’ BCS (Table 8). In Brazil, BMI and the same risk factors were evaluated for dog owners, and similarly, no statistical relationships were found with the dogs’ BCS [1]. On the other hand, according to Loftus and Wakshlag [3], the relationship between the obesity of owners and their pets is better described for dogs than for cats. A study on the association between the BMI of owners and the overweight of their dogs and cats was conducted in the Netherlands, with a significant positive correlation found only for dogs [30].

**Table 8:** Distribution of body condition score (BCS) frequencies according to the anthropometric measurements of cat owners in Goiânia, Brazil.

Characteristics	Underweight (BCS 1–4)		Ideal (BCS 5)		Overweight (BCS 6–7)		Obese (BCS 8–9)		Total			p-value*
	N	%	N	%	N	%	N	%	N		%	
**Owner’s body mass index**												
Underweight (<18.5)	2	(12.5)	9	(56.2)	3	(18.8)	2	(12.5)	16	(8.5)		0.800
Eutrophic (18.6–24.9)	27	(31.8)	35	(41.2)	17	(20.0)	6	(7.0)	85	(45.2)		
Overweight (25.0–29.9)	20	(36.4)	20	(36.4)	12	(21.8)	3	(5.4)	55	(29.3)		
Obese (≥30.0)	8	(25.0)	13	(40.6)	6	(18.8)	5	(15.6)	32	(17.0)		
**Owner waist/hip ratio**												
High risk	25	(37.9)	25	(37.9)	11	(16.6)	5	(7.6)	66		(35.1)	0.083
Moderate risk	27	(32.5)	34	(41.0)	17	(20.5)	5	(6.0)	83		(44.2)	
Low risk	5	(12.8)	18	(46.1)	10	(25.7)	6	(15.4)	39		(20.7)	
**Owner waist/height**												
At risk	31	(36.0)	34	(39.5)	14	(16.3)	7	(8.2)	86		(45.7)	0.389
No at risk	26	(25.5)	43	(42.2)	24	(23.5)	9	(8.8)	102		(54.3)	
**Owner abdominal circumference**												
At risk	48	(31.8)	63	(41.7)	29	(19.2)	11	(7.3)	151		(80.3)	0.497
No at risk	9	(24.3)	14	(37.9)	9	(24.3)	5	(13.5)	37		(19.7)	

BCS – Body condition score; *p value obtained by the Kruskal-Wallis test.

The underestimation of the animals’ body condition score (BCS) by their owners has been described as a risk factor for the development of overweight and obesity in cats [6,10,12,17]. According to Cave et al. [12], from a feline population health perspective, the association between obesity and the owner’s perception of their cat’s body condition suggests that more attention should be directed toward educating owners about the ideal body condition of a cat, rather than focusing educational efforts on changing feeding patterns or types of food for cats. Consistent with the literature [31], our results showed only a slight agreement (kappa = 0.177; p < 0.001; Table 9) between the cats’ BCS and the owners’ perceptions of these BCS. Interestingly, 71.1% and 75.0% of owners underestimated the BCS of overweight or obese cats, respectively (Table 9), classifying overweight cats as ideal (68.5%) and obese cats as overweight (50%) (Table 9).

**Table 9:** Comparison of agreement between body condition scores (BCS) determined by owner and veterinarians in a population of owned cats in Goiânia, Brazil.

Owner perception of the cats’ BCS	Underweight (BCS 1–4)		Ideal (BCS 5)		Overweight (BCS 6–7)		Obese (BCS 8–9)		p-value*	K^#^
	N	%	N	%	N	%	N	%		
Underweight	9	(15,8)	1	(1,3)	1	(2,6)	0	(0,0)	<0,001	0,177
Ideal	45	(78,9)	66	(85,7)	26	(68,5)	4	(25,0)		
Overweight	3	(5,3)	10	(13,0)	10	(26,3)	8	(50,0)		
Obese	0	(0,0)	0	(0,0)	1	(2,6)	4	(25,0)		
Total	57	(100)	77	(100)	38	(100)	16	(100)		

BCS – Body Condition Score; *p value obtained by the kappa test; ^#^K correspondent to kappa test (inter-rater agreement).

Despite being conducted with strict methodological criteria, this study had some limitations. Visits to the households were made during business hours, which likely increased the number of households with absent residents that were not sampled, as well as the sampling of owners who worked from home, were retired, or unemployed. Another limitation relates to the behavior of domestic cats. Many animals were not found by their owners at the time of the visit or were aggressive and did not allow the necessary assessments to be conducted. Furthermore, due to defensive behavior or fear of being judged, some owners may have answered questions about their own lifestyle habits or the management of their pets by stating what they believed to be correct, rather than reflecting the reality, causing unintended bias. Despite the small limitations inherent in applying questionnaires in field research, our results consistently provide, for the first time, an epidemiological diagnosis of obesity in domestic cats in a metropolitan area in Latin America.

## Conclusions

The prevalence of feline overweight and obese in Goiânia, Brazil, was estimated at 28.7%, with 20.2% of the cats being overweight and 8.5% obese. Factors associated with the development of overweight and obesity included the cat’s age (between 7 and 10 years), low physical activity, living in apartment, the owner’s education level (from high school to postgraduate), and the owner working outside the home. The presence of five or more cats in the same household, the location of the household in specific areas of the city, and the owner reporting that their own food intake is normal or excessive were protective factors against the development of overweight and obesity in the studied cat population. The BCS of overweight or obese cats was underestimated by 71.1% and 75%, respectively. This was the first study to provide important insights into the prevalence and risk factors of feline obesity in a metropolitan area of Latin America.

## References

[1] Porsani MYH, Teixeira FA, Oliveira VV, Pedrinelli V, Dias RA, German AJ, et al. Prevalence of canine obesity in the city of São Paulo, Brazil. Sci Rep. 2020;10(1):14082. doi: 10.1038/s41598-020-70937-8 32826948 PMC7442815

[2] Godfrey H, Morrow S, Abood SK, Verbrugghe A. Identifying the target population and preventive strategies to combat feline obesity. J Feline Med Surg. 2024;26(2):1098612X241228042. doi: 10.1177/1098612X241228042 38415669 PMC10911305

[3] Loftus JP, Wakshlag JJ. Canine and feline obesity: a review of pathophysiology, epidemiology, and clinical management. Vet Med (Auckl). 2014;6:49–60. doi: 10.2147/VMRR.S40868 30101096 PMC6067794

[4] Ludwig DS, Sørensen TIA. An integrated model of obesity pathogenesis that revisits causal direction. Nat Rev Endocrinol. 2022;18(5):261–2. doi: 10.1038/s41574-022-00635-0 35136206

[5] Saavedra C, Pérez C, Oyarzún C, Torres-Arévalo Á. Overweight and obesity in domestic cats: epidemiological risk factors and associated pathologies. J Feline Med Surg. 2024;26(11). doi: 10.1177/1098612X241285519 39560296 PMC11577473

[6] Allan FJ, Pfeiffer DU, Jones BR, Esslemont DH, Wiseman MS. A cross-sectional study of risk factors for obesity in cats in New Zealand. Prev Vet Med. 2000;46(3):183–96. doi: 10.1016/s0167-5877(00)00147-1 10913803

[7] Russell K, Sabin R, Holt S, Bradley R, Harper EJ. Influence of feeding regimen on body condition in the cat. J Small Anim Pract. 2000;41(1):12–7. doi: 10.1111/j.1748-5827.2000.tb03129.x 10713977

[8] Lund E, Armstrong P, Kirk C a., Klausner JS. Prevalence and risk factors for obesity in adult dogs from private US veterinary practices. The Journal of Applied Research in Veterinary Medicine. 2006;4:177–86.

[9] McGreevy P, Thomson P, Pride C, Fawcett A, Grassi T, Jones B. Overweight or obese cats presented to Australian veterinary practices: risk factors and prevalence. 2008. 10.1136/vr.156.22.695 15923551

[10] Colliard L, Paragon B-M, Lemuet B, Bénet J-J, Blanchard G. Prevalence and risk factors of obesity in an urban population of healthy cats. J Feline Med Surg. 2009;11(2):135–40. doi: 10.1016/j.jfms.2008.07.002 18774325 PMC10832791

[11] Courcier EA, Mellor DJ, Pendlebury E, Evans C, Yam PS. An investigation into the epidemiology of feline obesity in Great Britain: results of a cross-sectional study of 47 companion animal practises. Vet Rec. 2012;171(22):560. doi: 10.1136/vr.100953 23081976

[12] Cave NJ, Allan FJ, Schokkenbroek SL, Metekohy CAM, Pfeiffer DU. A cross-sectional study to compare changes in the prevalence and risk factors for feline obesity between 1993 and 2007 in New Zealand. Prev Vet Med. 2012;107(1–2):121–33. doi: 10.1016/j.prevetmed.2012.05.006 22703979

[13] Corbee RJ. Obesity in show cats. J Anim Physiol Anim Nutr (Berl). 2014;98(6):1075–80. doi: 10.1111/jpn.12176 24612018

[14] Wall M, Cave NJ, Vallee E. Owner and Cat-Related Risk Factors for Feline Overweight or Obesity. Front Vet Sci. 2019;6:266. doi: 10.3389/fvets.2019.00266 31482097 PMC6709657

[15] Chiang C-F, Villaverde C, Chang W-C, Fascetti AJ, Larsen JA. Prevalence, Risk Factors, and Disease Associations of Overweight and Obesity in Dogs that Visited the Veterinary Medical Teaching Hospital at the University of California, Davis from January 2006 to December 2015. Top Companion Anim Med. 2022;48:100640. doi: 10.1016/j.tcam.2022.100640 35104633

[16] Mendes-Junior AF, Passos CB, Gáleas MAV, Secchin MC, Aptekmann KP. Prevalência e fatores de risco da obesidade felina em Alegre-ES, Brasil. Sem Ci Agr. 2013;34(4). doi: 10.5433/1679-0359.2013v34n4p1801

[17] Alves RS, Barbosa RCC, Gheren MW, Silva L da, Souza HJM de. Frequência e fatores de risco da obesidade em uma população de gatos domésticos no Rio de Janeiro. BJVM. 2017;39(1):33–45. doi: 10.29374/2527-2179.bjvm0081

[18] Machado BS, Bruno CE, Silva DI, Barth JC, Santos LP, Alves MS, et al. An overweight/obesity survey among dogs and cats attended at a veterinary teaching hospital during the second year of the COVID-19 pandemic. Arq Bras Med Vet Zootec. 2022;74(6):999–1006. doi: 10.1590/1678-4162-12696

[19] IBGE. Censo 2010. 2010. https://cidades.ibge.gov.br/brasil/go/goiania/pesquisa/23/25207?tipo=ranking

[20] MINISTÉRIO DA SAÚDE. Guia alimentar para a população brasileira. 2nd ed. Brasilia: Ministério da Saúde. 2014.

[21] Laflamme DP. Development and validation of a body condition score for cats: A clinical tool. Feline Pract. 1997;24:13–8.

[22] Quimby J, Gowland S, Carney HC, DePorter T, Plummer P, Westropp J. 2021 AAHA/AAFP Feline Life Stage Guidelines*. Journal of the American Animal Hospital Association. 2021;57(2):51–72. doi: 10.5326/jaaha-ms-7189

[23] World Health Organization WHO. Obesity and overweight. https://www.who.int/en/news-room/fact-sheets/detail/obesity-and-overweight. 2017.

[24] Huang K-C, Lin W-Y, Lee L-T, Chen C-Y, Lo H, Hsia H-H, et al. Four anthropometric indices and cardiovascular risk factors in Taiwan. Int J Obes Relat Metab Disord. 2002;26(8):1060–8. doi: 10.1038/sj.ijo.0802047 12119571

[25] Tarkosova D, Story MM, Rand JS, Svoboda M. Feline obesity - prevalence, risk factors, pathogenesis, associated conditions and assessment: a review. Vet Med. 2016;61(6):295–307. doi: 10.17221/145/2015-vetmed

[26] Foreman-Worsley R, Blackwell E, Finka LR, Skillings E, McDonald JL. Long-term effect of neutering age on body condition score and bodyweight in domestic cats. Vet Rec. 2025;196(12):e5433. doi: 10.1002/vetr.5433 40390198 PMC12180291

[27] Teng KT, McGreevy PD, Toribio J-ALML, Dhand NK. Positive attitudes towards feline obesity are strongly associated with ownership of obese cats. PLoS One. 2020;15(6):e0234190. doi: 10.1371/journal.pone.0234190 32584825 PMC7316328

[28] Rowe E, Browne W, Casey R, Gruffydd-Jones T, Murray J. Risk factors identified for owner-reported feline obesity at around one year of age: Dry diet and indoor lifestyle. Prev Vet Med. 2015;121(3–4):273–81. doi: 10.1016/j.prevetmed.2015.07.011 26265631

[29] Pereira L. Desigualdades socioespaciais de Goiânia-Go: análise com base nos setores censitários subnormais. Universidade Federal de Minas Gerais. 2019.

[30] Nijland ML, Stam F, Seidell JC. Overweight in dogs, but not in cats, is related to overweight in their owners. Public Health Nutr. 2010;13(1):102–6. doi: 10.1017/S136898000999022X 19545467

[31] Teixeira FA, Queiroz MR, Oba PM, Olivindo RFG, Ernandes MC, Duarte CN, et al. Brazilian owners perception of the body condition score of dogs and cats. BMC Vet Res. 2020;16(1):463. doi: 10.1186/s12917-020-02679-8 33246455 PMC7694915

