# Supplementary material for: Household survey on prevalence and risk factors for obesity in owned cats from Central Brazil
Source: PLoS One. 2026 Jan 7;21(1):e0337397. doi: 10.1371/journal.pone.0337397 (PMC12779077; doi:10.1371/journal.pone.0337397)
Supplement: Attachment — Submitted filename: Response to Reviewers.docx [file pone.0337397.s002.docx]

**Response to Reviewers**

**Manuscript ID**: PONE-D-25-41354
**Title**: Household Survey on Prevalence and Risk Factors for Obesity in Owned Cats from Central Brazil

**Dear Editor and Reviewer,**

We thank you for your careful evaluation of our manuscript and for the constructive feedback provided. We sincerely appreciate the opportunity to revise our work and respond to the comments raised during the review process.

Please find below our point-by-point response.

### **Reviewer #1**

**Comment:**
“Since there was a statistical difference in the stratification of owners according to household income, it would be important to specify which currency was used in this stratification. I believe that the Brazilian currency may have been converted into US dollars; however, this was not made clear.”

**Response:**
Thank you for your helpful suggestion. We agree that it is important to clarify the currency used in the income stratification.

We have revised the manuscript to explicitly state that household income, originally reported in Brazilian reais (BRL), was converted into US dollars (USD) for reporting and analysis purposes. This information has been added to the “Materials and Methods” section under the subsection “Data Collection and Categorization of Cats and Owners”.

**Change made in the manuscript (page 6, lines 129–131):**

“For the purpose of stratifying owners by household income, amounts in the Brazilian currency, the Brazilian real, were converted into US dollars.”

We believe this amendment addresses the reviewer’s concern and improves the clarity of the manuscript.

We thank you again for your time and consideration. We hope our revised version meets the journal’s standards and look forward to your favorable response.

Sincerely,
**Dr. Danilo Conrado Silva**
on behalf of all co-authors
